# The anterior temporal cortex is a primary semantic source of top-down influences on object recognition

**DOI:** 10.1016/j.cortex.2016.03.007

**Published:** 2016-06

**Authors:** Rocco Chiou, Matthew A. Lambon Ralph

**Affiliations:** The Neuroscience and Aphasia Research Unit (NARU), School of Psychological Sciences, University of Manchester, England, UK

**Keywords:** Anterior temporal lobe, TMS, Perception, Semantic memory, Representational geometry

## Abstract

Perception emerges from a dynamic interplay between feed-forward sensory input and feedback modulation along the cascade of neural processing. Prior knowledge, a major form of top-down modulatory signal, benefits perception by enabling efficacious inference and resolving ambiguity, particularly under circumstances of degraded visual input. Despite semantic information being a potentially critical source of this top-down influence, to date, the core neural substrate of semantic knowledge (the anterolateral temporal lobe – ATL) has not been considered as a key component of the feedback system. Here we provide direct evidence of its significance for visual cognition – the ATL underpins the semantic aspect of object recognition, amalgamating sensory-based (amount of accumulated sensory input) and semantic-based (representational proximity between exemplars and typicality of appearance) influences. Using transcranial theta-burst stimulation combined with a novel visual identification paradigm, we demonstrate that the left ATL contributes to discrimination between visual objects. Crucially, its contribution is especially vital under situations where semantic knowledge is most needed for supplementing deficiency of input (brief visual exposure), discerning analogously-coded exemplars (close representational distance), and resolving discordance (target appearance violating the statistical typicality of its category). Our findings characterise functional properties of the ATL in object recognition: this neural structure is summoned to augment the visual system when the latter is overtaxed by challenging conditions (insufficient input, overlapped neural coding, and conflict between incoming signal and expected configuration). This suggests a need to revisit current theories of object recognition, incorporating the ATL that interfaces high-level vision with semantic knowledge.

## 1 Introduction

Traditional notions of object recognition have been unidirectional and hierarchical – neural processing of visual objects courses through subregions of the striate and extrastriate cortices, traversing in a feed-forward fashion, and finally culminates in the formation of object representation in the ventral temporal cortex. Evidence of feedback modulation accumulated over the last decade, however, has made the reciprocal nature of the visual system apparent (Gilbert & Li, 2013). It is now established that endogenous influences, such as attention, expectation, and memory, facilitate perception via prioritising signals and constraining perceptual interpretations. Expectation based on conceptual/semantic knowledge, in particular, has been demonstrated to exert a striking impact on object recognition (for review, see Panichello et al., 2012, Trapp and Bar, 2015). For instance, a fleeting glimpse of a stringy object in a yacht deck scene is more likely to be recognised as a rope rather than a snake, because semantic knowledge informs us about the object's possible location and the items it would be juxtaposed with. Despite ample behavioural evidence, we still have limited understanding about how such semantically-based top-down modulation arises in the brain. The orbitofrontal cortex (OFC) has been suggested as the neural source of feedback messages to visual cortices (Bar et al., 2006). According to the predictive feedback account, the OFC creates coarse representations using low spatial-frequency visual information, which is projected back to the inferior temporal visual cortex to enhance compatible signals and mitigate those incompatible (Trapp & Bar, 2015). In addition to the OFC and posterior visual areas, however, it is unclear whether the neural substrates of semantic knowledge are directly involved in this modulatory process. This is especially surprising given the clear involvement of semantic information in object processing.

In the present study, we address this issue by investigating the possible role of the anterior temporal lobe (ATL) in object recognition. Converging evidence from neuroimaging, neurostimulation and neuropsychological research has indicated that the ATL serves as a representational hub for disparate streams of modality-based information to merge and transcend into modality-invariant context-independent concepts (for review, see Lambon Ralph, 2014, Patterson et al., 2007). The importance of the ATL in semantic representation is perhaps most strikingly and convincingly demonstrated in the deficits of patients with semantic dementia (SD). With atrophy/hypo-metabolism centred mostly on ventrolateral aspects of the ATL, these patients are impaired at various semantic-oriented tasks involving verbal (words) or non-verbal (images, sounds, etc.) materials and requiring verbal (naming) or non-verbal (e.g., gesturing to illustrate proper object use) responses. Intriguingly, albeit somewhat overlooked in the literature, SD patients show deficits hinting at a difficulty in processing visual stimuli when the viewing conditions are challenging. For instance, Cumming, Patterson, Verfaellie, and Graham (2006) adopted a visual matching paradigm and asked SD patients to make same-different judgements on sequentially-presented letters, objects, or meaningless shapes. For meaningful stimuli (letters and objects), the patients showed a striking decline in visual matching performance when stimuli were presented briefly (67 msec) and backward-masked but exhibited perfect accuracy when stimuli were shown long enough (200 msec) or not degraded by masking. For meaningless stimuli (irregular geometric shapes), however, the patients showed ceiling level accuracy, unaffected by brief presentation and masking. Moreover, the nature of the SD patients' deficits stood in marked contrast with those of patients with pure alexia (PA, caused by posterior artery stroke leading to ventral occipitotemporal damage). Whereas PA patients performed poorly irrespective of meaning and viewing condition, SD patients were selectively impaired for meaningful stimuli presented briefly and subsequently masked. These clear-cut differences suggest that ATL atrophy can negatively impact on the ability to process meaningful items effectively via reduced top-down semantic support for visual identification.

Could the ATL-based semantic hub be a key neurocognitive component in the physiological foundation of ‘top-down vs. bottom-up’ neural dynamics? We answered this question by using continuous theta-burst stimulation (cTBS) combined with a novel visual discrimination paradigm in which we pitted the amount of accumulated visual evidence available (exposure duration) against two semantic properties of objects. Specifically, we temporarily disrupted processing in this representational hub by targeting cTBS at the ventrolateral aspect of the left ATL, a region crucial for semantic processing and accessible to stimulation (Chiou et al., 2014, Pobric et al., 2009), and compared ATL against vertex stimulation, a well-established control site not involved in most high-level cognitive processing (Sandrini, Umilta, & Rusconi, 2012). Based on three lines of inquiry, we independently manipulated three different experimental factors to probe the extent of ATL involvement in visual object perception:

(i) The first factor concerns the impact of visual duration. Previous neuroimaging explorations of visual word recognition by Price et al. showed that, whereas longer visual presentation induced more posterior occipital activity (due presumably to greater visual stimulation), brief presentation triggered greater activation spread across anterior temporal and prefrontal cortices (Mechelli et al., 2000, Price et al., 1994). This implies up-regulation of the ATL system to compensate insufficient input to the perceptual system, consistent with the clinical observation on SD patients (Cumming et al., 2006), and also hints that applying cTBS to tamper with ATL processing would be particularly harmful for brief displays.
(ii) The second factor concerns the representational “geometry” (Kriegeskorte & Kievit, 2013). Kriegeskorte et al. quantified representational distances between objects of different categories by rendering their dissimilarity of inferior-temporal (IT) response patterns onto a dendrogram, visualising the divergence of neural coding as ‘geometric proximity’ (Carlson et al., 2014a, Kriegeskorte et al., 2008). With this visualisation, an intriguing pattern concerning the relations between categories becomes evident – although plants and animals are both living entities, plants are represented more similarly to non-living artefacts (belonging to the same cluster tree), whereas animals are much more tightly packed and are represented distinctly from plants and artefacts (a separable cluster). Resembling this neural coding similarity, there is a noticeable parallelism in the taxonomic structure of objects that results from similarity analyses of detailed semantic feature databases (Carlson et al., 2014b, Cree and McRae, 2003, Dilkina and Lambon Ralph, 2013, Garrard et al., 2001, Hoffman and Lambon Ralph, 2013, Rogers et al., 2004). This implies that disentangling items from two representationally overlapping categories (thus greater resemblance in IT coding) would necessitate more top-down support.
(iii) The third factor concerns the degree to which an exemplar resembles the typical appearance of its category (e.g., spinach epitomises a canonically defined ‘vegetable’ whereas turnip is less typical). Previous research has demonstrated that ATL damage makes SD patients highly sensitive to conceptual typicality (Lambon Ralph et al., 2010, Mayberry et al., 2011, Rogers et al., 2015), including the typicality of canonical object colour (Rogers et al., 2007, Rogers et al., 2015). Specifically, the patients' performance on object classifications were disproportionally impaired when encountering atypical-looking targets or foils that possessed typical features of the target category. This suggests that, to accept atypical targets and correctly reject pseudo-typical foils, the ATL is a key substrate for counteracting superficial sensory similarity (which misleads responses) and extracting conceptual coherence (which defines the semantic criterion that demarcates targets from foils).

Based on the known characteristics of the ATL discussed above, we predicted that targeting cTBS to this region would particularly impair cognitive performance when semantic knowledge is most needed for bolstering visual recognition (i.e., brief presentation, similar neural representation, and violation of typicality). This may lead to deterioration of performance (declined accuracy/prolonged processing time) and weaken the behavioural signature that indexes normal neurocognitive functioning. To pre-empt the results, we found that disrupting the neural processing of the left ATL was particularly detrimental to object identification under challenging perceptual and conceptual conditions. These perceptual and conceptual factors also intricately interacted with each other, making TMS-induced deterioration most pronounced when the ‘triple whammy’ occurred. In the Discussion, we elaborate on the implication of these results for theories of object recognition and the reciprocity of the visual system in general.

## 2 Material and methods

### 2.1 Participants

Twelve volunteers (6 females, age: 26 ± 5 years) gave informed consent before participation. All reported using their right hand as the dominant hand to write and carry objects, thus all classified right-handed. All had normal (or corrected-to-normal) vision, completed safety screening for TMS and MRI before the experiment, and reported no history of neurological disease/injury or psychiatric condition. This study was reviewed and approved by the local research ethics committee.

### 2.2 Apparatus

In the first session, we acquired a high resolution T1-weighted structural image for each participant using a 3T Philips Achieva scanner and an 8-element head-array coil, with in-plane resolution of .94 mm and slice thickness of .9 mm. In the second and third session, we conducted the transcranial magnetic stimulation (TMS) experiments. Visual stimuli were presented using MATLAB with Psychophysics (Brainard, 1997, Pelli, 1997) on a computer monitor (29 × 39.5 cm; 75 Hz refresh rate; 1024 × 768 resolution). Participants' head position was stabilised with a chin-rest, keeping a viewing distance of 57 cm from the screen. We applied brain stimulation using a Magstim Super Rapid^2^ system with a figure-of-eight coil (70 mm). Positioning of the stimulation coil was guided using a frameless stereotaxic neuronavigation system (Brainsight 2, Rogue Research Inc.) paired with Polaris Vicra sensor camera and infrared-emitting markers that allow on-the-fly calibration during stimulation (see below for details of TMS protocol).

### 2.3 Design

We used a 2 × 2 × 2 × 2 within-participant factorial design, with Stimulation Site (region of interest: the left anterolateral temporal cortex, control site: the vertex), Representational Proximity (plant target with artefact foil, animal target with artefact foil; based on previous research, when occipitotemporal representations are rendered graphically onto a virtual space, plants/animals are close/distant to artefacts; see the Stimuli section for details), Stimulus Duration (40 msec, 600 msec), and Typicality of Target (typical, atypical) as repeated-measure factors. In separate sessions, we stimulated one of the two cortical regions. Participants were asked to discriminate targets from foils, recognising exemplars that belong to the target category (performed in separate blocks of trials). We counterbalanced the order of stimulation site (in separate sessions) and target category (in alternating blocks) across participants, with different durations and typicality trials shuffled within a block.

### 2.4 Stimuli

We constructed a unique set of object images. These materials were created based on relevant neuroimaging and neuropsychological evidence. First, as noted above, analysis for multiple-voxel patterns of occipitotemporal cortex has revealed that plant and artefact exemplars elicit resembling patterns of neural activation, which forms a cluster ‘geometrically’ more distant to the cluster of animals in representational space (Kriegeskorte et al., 2008). Consistent with this neural similarity evidence, analyses based on large feature-listing studies have also obtained the same clustering structure, supporting its reliability across methods (e.g., Dilkina and Lambon Ralph, 2013, Rogers et al., 2004). Second, exemplar-specific knowledge is most eroded by ATL atrophy whereas category-generic concepts are better preserved. For example, in object-colour matching, SD patients often erroneously selected green for fruit and vegetables, and brown for animals (Rogers et al., 2007, Rogers et al., 2015).

**Fig. 1:**
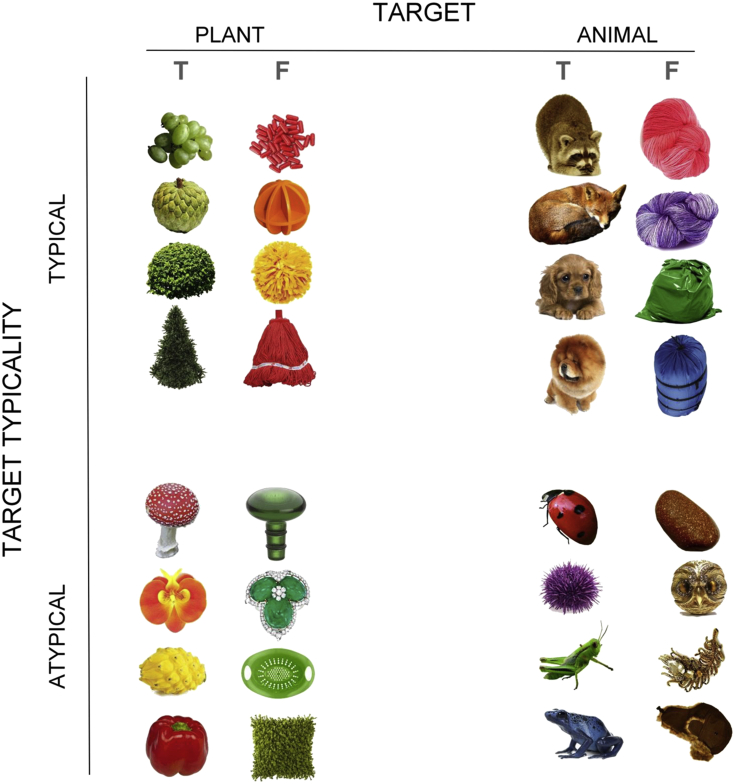
Example stimuli from each of the four conditions (plant-artefact, animal-artefact × typical, atypical). T: target; F: foil.

Based on these data, we constructed 320 object images, comprising the factorial combination of representational distance between items (near: plant-artefact, far: animal-artefact), typicality of target (typical, atypical), and 80 exemplars in each condition (see Fig. 1 for examples). As illustrated in Fig. 1, each target was coupled with an artefact foil, and the items were separated into four divisions. In the typical target condition, all target exemplars had a colour characteristic of their domain (green for plant, brown for animal), and their paired foils had a colour unusual for the target's category. Conversely, in the atypical target condition, the targets' colours were less common for their category whereas the foils' colours were typical. With careful selection we ensured that each colour was equally probable to appear in the typical and atypical conditions so that any difference between the two conditions cannot be attributed to probabilistic frequency of colours. We avoided using semantically-related objects in a pair, such as a canary (target) with a cage (foil). To ensure un-relatedness and the absence of any systematic difference between conditions, we asked five volunteers (none participated in the TMS experiment) to rate the degree of association for each pair of objects on a 5-point scale (1: completely unrelated, 5: intimately related). Results showed that relatedness rating approached the floor level in all of the four conditions (mean ± 1 SD – typical plant: 1.29 ± .43, atypical plant: 1.22 ± .25, typical animal: 1.17 ± .20, atypical animal: 1.34 ± .43) and did not significantly differ across conditions (*F*_3,156_ = 1.87, *p* = .14, *n.s*.).

**Fig. 2:**
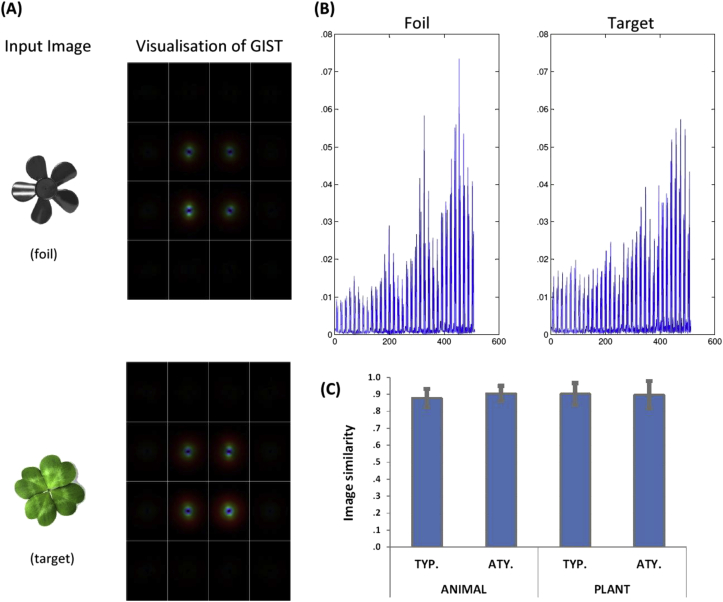
(A) a pair of target and foil stimuli and the visualisation of their GIST descriptor; (B) the distribution of the 512 GIST descriptor values for each image; (C) The mean score of target-foil image similarity for the four conditions, based on the GIST algorithmic verification. On the scale of the *y*-axis (0–1), a higher value represents greater visual similarity between targets and foils. Error bar: 1 SEM.

In addition, targets and foils were carefully selected with respect to their visual appearances. In each pair of objects, the artefact foil was picked to match its accompanying target in overall configuration and image size. We selected manmade items with the visual properties that most biological entities possess (e.g., curviness, symmetry, etc.) and avoided using those consist of straight lines and sharp angles that typify artefacts. To ascertain the visual similarity between targets and foils, we computed and compared their low-level visual properties using the extensively applied GIST descriptor algorithm (Oliva & Torralba, 2001). For each individual image, we first passed it through a series of Gabor filters across eight orientations and four spatial frequencies, giving 32 filtered images. These were subsequently rendered along a 4 × 4 grid to derive a GIST descriptor (a vector of 512 values), which characterised an image in terms of its spatial frequencies and orientations present at different locations scattered across the image (see Fig. 2A and B for illustrations). In the final step of this analysis pipeline, we computed image similarity for each pair of object by comparing their GIST descriptors; the scores ranged from zero to one, with higher values denoting greater visual similarity. Comparison of the image similarity scores across conditions showed that the degree of similarity approached ceiling in all of the four conditions (range: .88–.90; Fig. 2C) and, importantly, there is no reliable difference in similarity score between conditions (*F*_3,156_ = 1.51, *p* > .21, *n.s.*). This indicates that our target and foil images were well-matched on visual statistics and that any difference between conditions, be it ‘typical vs. atypical’ or ‘plant vs. animal’, cannot be explained by pictorial factors.

### 2.5 Psychophysical procedure

**Fig. 3:**
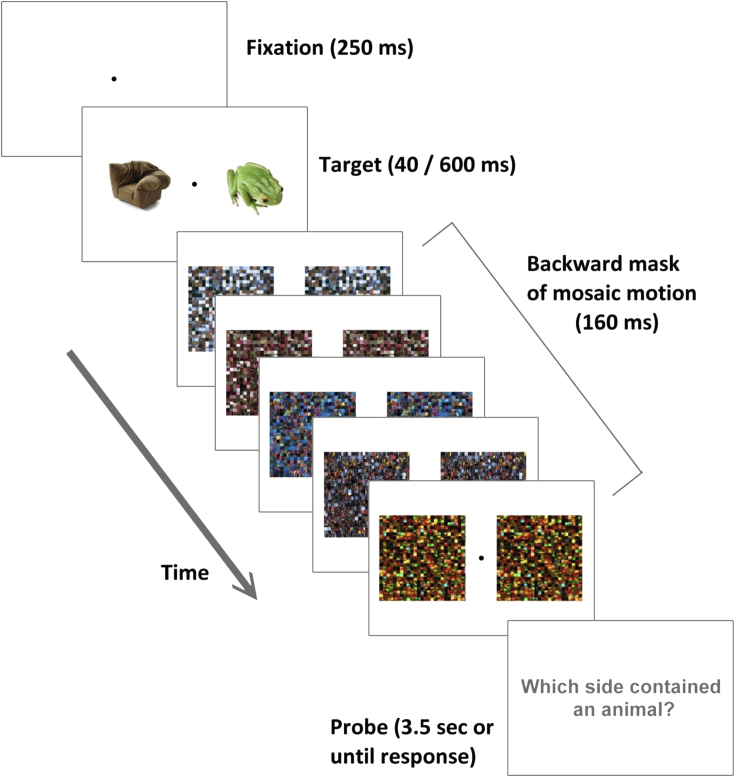
Timeline illustration of events in a trial. Note that during the 160-msec backward masking period the motion stimuli refreshed at 75 Hz, rapidly changing the colours of the mosaic stimuli 12 times. For simplicity, here in the Figure we only present 5 frames.

As Fig. 3 illustrates, each trial began with a black fixation dot on a white background (250 msec), followed by two object images, situated 7.5° to the left/right of the central point, presented for 40 msec or 600 msec. Following the target images, two square patterns of mosaic-motion (backward masking) were presented that subtended diagonally 20.5° and consisted of assemblages of coloured cells (25 × 25 grid). The motion rapidly refreshed the colour of each cell at the rate of 75 Hz for 160 msec. A response probe was presented subsequent to the masking motion, querying which side of the screen contained the target object (plant/animal, shown in separate blocks; probe duration: 3.5 sec or until response). Participants had to recognise the objects and pressed a designated button using their left/right index finger to indicate the target. There was a 250-msec interval between trials during which participants viewed a blank screen.

There were four blocks of 82 trials in each session (2 blocks of each task), yielding 40 trials in each experimental condition. Each block consisted of 80 target-present trials that required a response and two target-absent trials in which only two artefacts were shown and participants were instructed to withhold response under this condition. The few target-absent trials were added to prevent habitual response or task strategy. The 160 target-foil pairs were randomly assigned into Set One and Set Two; half of the participants viewed objects of Set One in the 40-msec condition and those of Set Two in the 600-msec condition, and for the remaining participants the Sets and presentation conditions were reversed. Each block consisted of an equal number of typical/atypical and 40-msec/600-msec trials, randomly intermingled. We counterbalanced all experimental parameters for the stimuli so that each individual stimulus, be it a target or a foil, was equally likely to be located on the left/right of the screen, responded to by the left/right hand, and presented in the 40-msec/600-msec condition. Prior to starting the co-registration procedure of the TMS protocol, we asked participants to complete two practise blocks of 10 trials.

Before carrying out the TMS experiments, we tested a group of six volunteers (none participated in the TMS study) to assess the impacts of the cognitive factors we manipulated when there was no perturbation to the brain. The outcome of this no-TMS pilot experiment ensured us that vertex stimulation was able to serve as a proper baseline that accorded with the performance under circumstances of no-TMS. See Footnote One for the pilot results.^1^

### 2.6 TMS procedure

We adopted an offline stimulation paradigm (i.e., participants received cTBS prior to the tasks and their performance was probed *immediately* following stimulation). This design avoids non-specific interference due to discomfort, noise, muscle twitches, and so on, relative to online paradigms (i.e., applying concomitant stimulation during task execution). This design had two additional advantages over the low-frequency (1 Hz) stimulation usually employed to test ATL functions (e.g., Pobric, Jefferies, & Lambon Ralph, 2007). Firstly, whereas 1 Hz TMS takes at least 10 min to complete, in the present study cTBS took only 1 min, minimising discomfort during stimulation. Secondly, compared to the short-lasting effect of 1 Hz TMS (which usually dissipates in 10 min; Sandrini et al., 2012), cTBS might be able to produce greater inhibitory impact in terms of magnitude and longevity and is suggested to be effective for probing high-level cognitive functions (although note previous demonstrations of the long-lasting effect were based on motor cortex stimulation eliciting motor-evoked potential; see Huang, Edwards, Rounis, Bhatia, & Rothwell, 2005).

We applied cTBS using a Magstim Rapid^2^ system and a 70-mm figure-of-eight induction coil. Stimulation was delivered to the targeted site in repeated trains of 300 bursts (3 magnetic pulses per burst; 50 Hz) with an inter-train-interval of 200 msec (5 Hz); the stimulation lasted for 60 sec, with a total number of 900 magnetic pulses. The strength of stimulation was set at 80% of resting motor threshold (RMT, the minimum stimulation intensity on the motor cortex that causes a visible finger twitch; to test individual RMT, we applied single-pulse stimulation to the left primary motor cortex; the value was defined as the minimum strength capable of eliciting visible twitches in the right abductor pollicis muscle on six out of ten contiguous trials). The averaged intensity of stimulation was 43 ± 2% of the stimulator maximum output (range: 40%–48%).

**Fig. 4:**
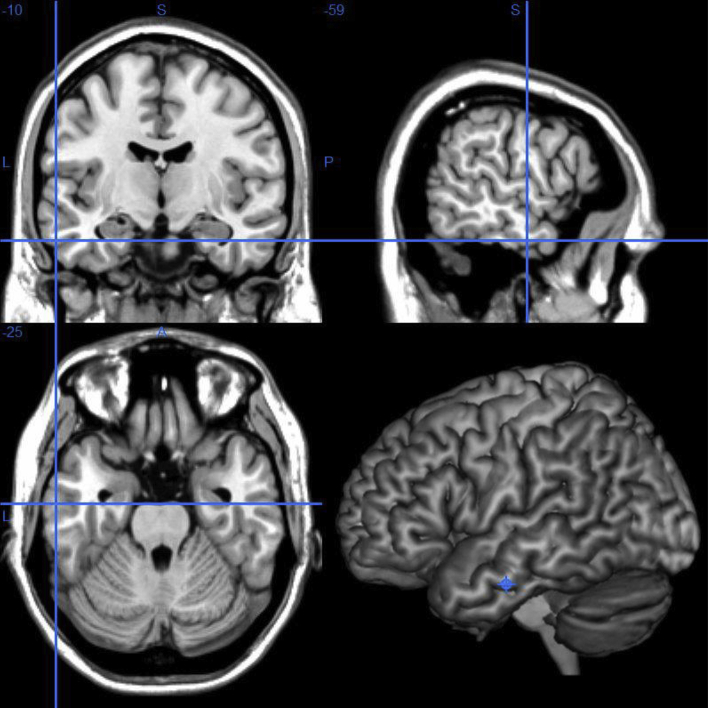
ATL stimulation sites pinpointed on the MNI cortical template.

Target sites for cTBS were localised individually based on T1-weighted MR structural scan and cerebral-scalpal co-registration. Neuroanatomical definitions for the ATL were based on a relevant functional neuroimaging study which explored the neural correlates of a representational semantic ‘hub’ where disparate streams of auditory and visual modality-based processing converge. We selected the peak activation of a ventral ATL cluster that showed modality-invariant responses when participants were engaged in semantic processing on visual and auditory stimuli (MNI coordinates: [−36 −9 −36]; Visser & Lambon Ralph, 2011). For each TMS participant, we normalised their structural image into the standardised space of MNI system using SPM8 (Wellcome Department of Imaging Neuroscience, London, U.K.) then converted the coordinates of our literature-defined ventral ATL site to derive the corresponding coordinates in each participant's anatomical native space. As the location of the directly converted ATL site was too ventral and medial to be accessed by stimulation on the scalp, we adjusted the coordinates based on individual anatomy, making it slightly more lateral to the original site and hence accessible to TMS. The averaged MNI coordinates of the ventral ATL across participants were [−59 ± 4, −10 ± 5, −25 ± 3] (see Fig. 4). The control site vertex was defined as the midpoint between each individual's nasion and inion, along the sagittal midline of the scalp.

Before the behavioural experiments, we performed a co-registration procedure mapping the cerebral site of TMS target of each session onto the corresponding point on the scalp using the Brainsight neuronavigation system, which tracked the coil's position during stimulation and allowed online adjustment to achieve precise positioning. For both sites, the coil was placed tangentially to the scalp with the handle pointing posteriorly (parallel to the rostral–caudal axis). For each individual, the TMS sessions were separated by at least 48 h, and performing the cognitive tasks (after TMS) took approximately 20 min.

## 3 Results

**Fig. 5:**
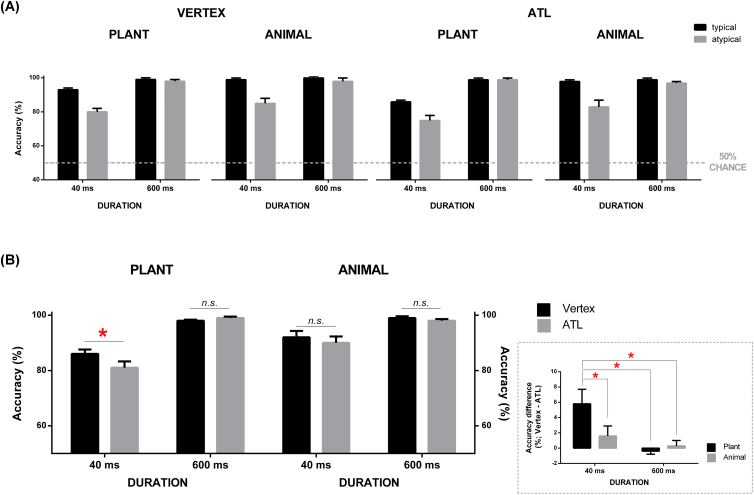
(A) Accuracy as a function of Stimulation Site (vertex vs. ATL), Representational Proximity (close: plant-artefact, distant: animal-artefact; for simplicity this is denoted ‘plant’ and ‘animal’ on the figure), Duration (40 msec, 600 msec), and Typicality (typical, atypical). (B) the significant three-way interaction (Stimulation Site × Representational Proximity × Duration), plotted as a function of accuracy. The inset boxes illustrate the accuracy difference between the two stimulation sites (vertex minus ATL), plotted as a function of Representational Proximity and Duration. Error bars represent +1 SEM. *p* < .05 *.

The mean accuracy for each condition is reported in Fig. 5A. Irrespective of typicality, performance was at ceiling in the 600-msec condition but it declined in the 40-msec condition with a more manifest drop for atypical targets. Closer scrutiny uncovered that ATL stimulation led to lower accuracy compared to the vertex; this reduction was most obvious for ‘plant targets and artefact foils’ displayed briefly while other conditions seemed relatively unaffected. This pattern was fully supported by the statistical analyses.

For accuracy, we undertook a four-way repeated-measure ANOVA, including within-participant factors of Stimulation Site (ATL, vertex), Representational Proximity (close: plant-artefact, distant: animal-artefact), Duration (40 msec, 600 msec), and Target Typicality (typical, atypical). Results revealed significant main effects of Stimulation Site (*F*_1, 11_ = 5.38, *p* = .04, *η*_p_^2^ = .32), Representational Proximity (*F*_1, 11_ = 17.40, *p* = .002, *η*_p_^2^ = .61), Duration (*F*_1, 11_ = 42.49, *p* < .001, *η*_p_^2^ = .79), and Typicality (*F*_1, 11_ = 33.21, *p* < .001, *η*_p_^2^ = .75). These factors also interacted with one another, including Stimulation Site × Duration (*F*_1, 11_ = 10.19, *p* = .009, *η*_p_^2^ = .48), Representational Proximity × Duration (*F*_1, 11_ = 12.82, *p* = .004, *η*_p_^2^ = .53), and Typicality × Duration (*F*_1, 11_ = 14.37, *p* = .003, *η*_p_^2^ = .56). Critically, there is a significant three-way interaction: Stimulation Site × Representational Proximity × Duration (*F*_1, 11_ = 7.82, *p* = .01, *η*_p_^2^ = .41). All other statistics were not significant (all *p*s > .15). Based on the highest-order significant three-way interaction, we conducted *a posteriori* comparisons to identify the origin of this effect (paired-sample *t*-test, examining how the effect of ‘vertex vs. ATL’ was differentially modulated by Representational Proximity and Duration). As Fig. 5B illustrates, object recognition was disproportionally disrupted by ATL stimulation when confronted with adjacently represented items shown briefly: ATL stimulation significantly worsened accuracy for the displays of ‘plant target and artefact foil’ presented briefly (81%) compared to identical stimuli and duration under vertex stimulation (87%, *p* = .01). By contrast, performance did not differ between the two stimulation sites in all other conditions (all *p*s > .26, *n.s.*), indicating the interference occurring under a specific combination of contextual factors – perturbing the ATL, brief display, and closely represented entities. Further analysis examining this interaction revealed that the magnitude of cTBS impact, indexed as the accuracy difference of vertex minus ATL, was significantly greater for ‘plant and artefact’ shown briefly than that in any other three conditions (see Fig. 5B inset box; all three *p*s ≤ .05; all three Cohen's *d*s ≥ .77, range: .77–1.56).

**Fig. 6:**
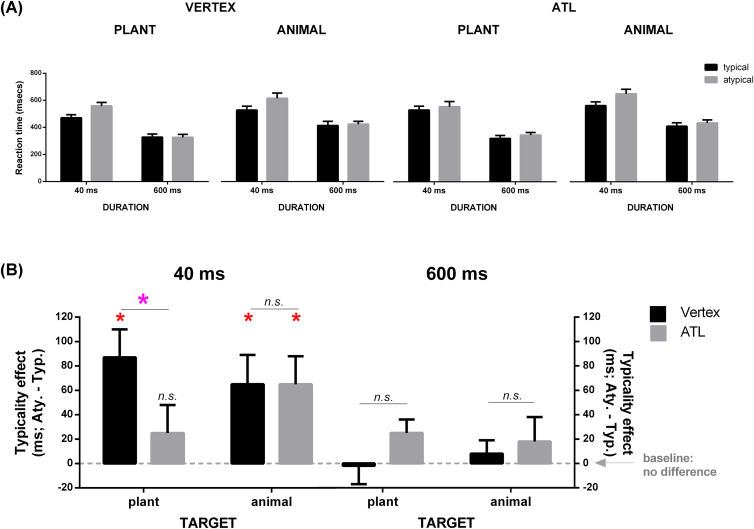
(A) Reaction time as a function of Stimulation Site (vertex vs. ATL), Representational Proximity (close: plant-artefact, distant: animal-artefact; for simplicity this is denoted ‘plant’ and ‘animal’ on the figure), Duration (40 msec, 600 msec), and Typicality (typical, atypical). (B) the significant four-way interaction (Stimulation Site × Representational Proximity × Duration × Typicality), plotted as a function of Typicality effect (atypical minus typical). Error bars represent +1 SEM. *p* < .05 *(red asterisk: the contrast of ‘typical vs. atypical’ displays; violet asterisk: the contrast of ‘ATL vs. vertex’).

The mean reaction times (RTs) of each condition are reported in Fig. 6A. As evident in the Figure, regardless of typicality, RTs were generally faster in the 600-msec condition than those in the 40-msec condition, with minimal difference between typical and atypical targets. Within the 40-msec condition, further examination revealed an obvious pattern in which atypical targets led to prolonged RTs relative to typical targets, implying a typicality effect. However, this effect dwindled in size when the stimuli were brief displays of ‘plant target and artefact foil’ following ATL stimulation. Again these patterns were corroborated by the formal statistical analyses.

Prior to analysis, we excluded errors (6.4%) and outliers (2.4%; RTs faster than 100 msec or slower than 3SD above the condition mean). Identical to the analysis of accuracy, we carried out a repeated-measure ANOVA with Stimulation Site, Representational Proximity, Duration, and Target Typicality as within-participant variables. We found significant main effects of Representational Proximity (*F*_1, 11_ = 11.29, *p* = .006, *η*_p_^2^ = .50), Duration (*F*_1, 11_ = 42.21, *p* < .001, *η*_p_^2^ = .79), and Typicality (*F*_1, 11_ = 16.62, *p* = .002, *η*_p_^2^ = .60). We also found a Representational Proximity × Duration interaction (*F*_1, 11_ = 33.20, *p* < .001, *η*_p_^2^ = .75). Most important, we obtained a significant four-way interaction involving *all* factors (*F*_1, 11_ = 8.65, *p* = .01, *η*_p_^2^ = .44; see Fig. 6B). Due to the complexity of the four-way interaction, we first conducted analysis of simple effect by Duration to dissect the pattern. Within the data of the 600-msec condition, neither the main effects of the remaining three factors reached significance nor did they interact (all *p*s > .21, *n.s.*). Results of the 40-msec condition showed striking differences: there were significant main effects of Representational Proximity (*F*_1, 11_ = 18.37, *p* = .001, *η*_p_^2^ = .62) and Typicality (*F*_1, 11_ = 12.56, *p* = .005, *η*_p_^2^ = .53). Pertinent to our interest, there was a significant Stimulation Site × Representational Proximity × Typicality interaction (*F*_1, 11_ = 5.86, *p* = .03, *η*_p_^2^ = .34; see the left half of Fig. 6B). Based on this significant interaction, we conducted *a posteriori* tests, exploring how the typicality effect (indexed as atypical RTs minus typical ones) was modulated by Stimulation Site and Representational Proximity. Consistent with initial visual inspection, we found that the typicality effect was immune to cTBS perturbation in every condition (all *p*s < .03; red asterisk, indicating significant slowing for atypical displays; note the comparison was *within* each condition, contrasting typical vs. atypical), except for the displays of ‘plant and artefact’ under ATL stimulation (*p* > .30, *n.s*., indicating cTBS wiping out the typicality effect). Further analysis showed that, whereas the size of the typicality effect did not differ between vertex and ATL for representationally distant pairs (animal and artefact; *p* > .97, *n.s*., indicating equivalent strength; note the comparison was *between* conditions, contrasting ATL vs. vertex), it significantly differed between the two sites for representationally close pairs (plant and artefact; *p* = .03, Cohen's *d* = .77; violet asterisk, indicating a significant difference in the strength of typicality effect *between* TMS sites), with the effect shrinking in magnitude following ATL stimulation.

## 4 Discussion

Although there is growing evidence that semantic knowledge benefits perception, surprisingly, we still have limited understanding as to *whether* and *how* a key neural underpinning of semantic processing – the ATL region – contributes to visual object identification. Using theta-burst stimulation combined with a novel visual identification paradigm, we established the necessity of the ATL in buttressing object recognition and, more importantly, discovered the specific circumstances in which the ATL contributes most to high-level vision. In accuracy, disrupting the left ATL deteriorated performance selectively for similarly-represented items displayed briefly. In RTs, ATL stimulation eradicated the otherwise robust advantage of objects that exemplified their category (typicality effect: shorter latency for typical items); like accuracy data, effects of cTBS occurred selectively for similarly-represented items shown briefly. With careful control over low-level pictorial properties and pair-wise target-foil relatedness, we ensured the effect cannot be driven by visual statistics and semantic association. By including both a control site (the vertex) and a control condition (600-msec display) that provided a baseline, our paradigm allowed ruling out non-specific effects of TMS and thus underscores the specificity of ATL contribution.

To delineate the dynamics between the ATL structure and various perception- and semantic-based factors, we first discuss the outcome of the vertex stimulation, which concords with the no-TMS pilot data. These results illustrate how the neural system normally behaves when there is no disruption/damage to the distributed network underpinning object recognition. With sufficient bottom-up information to the visual system (600-msec display), object recognition was highly accurate and efficient, achieving ceiling performance that obscures any contribution of top-down influences. However, when visual input was reduced by shortened exposure, we observed a clear indicator of top-down support; while performance overall was compromised by insufficient input, the decline was less severe for typical items. This meshes closely with psychophysical findings in the visual search literature (Dunovan et al., 2014, Maxfield et al., 2014, Vickery et al., 2005): when one searches for exemplars of a target category, prior knowledge sets up a ‘template’ that encompasses most frequent features (e.g., expecting something greenish for plant). Items that match this template are prioritised and enhanced, reducing reliance on bottom-up input. By contrast, items partially mismatching the template require more accumulation of perceptual evidence, which demands longer exposure durations. This ‘top-down vs. bottom-up’ synergy is embodied in the typicality effect during brief displays.

When ATL stimulation perturbs the semantic component of object recognition system, we observed a breakdown of the synergistic operation between top-down and bottom-up forces. Interestingly, perturbing the ATL also augmented the difference between adjacently-vs. distantly-represented pairs – under brief displays, closely-represented items were most vulnerable to ATL stimulation. Previous work on the representational organisation of semantic entities primarily focused on the IT cortex (for review, see Kriegeskorte & Kievit, 2013). As mentioned, compared to animals, fruit/vegetables evoked more similar patterns of IT activity to those of artefacts (Kriegeskorte et al., 2008). Recent studies have further shown that RT for object categorisation can be predicted using the patterns of IT neural representational similarity (Carlson, Ritchie, et al., 2014) and that this neural-to-behavioural predictability peaked at a narrow time-window (120–240 post-stimulus) during which animate and inanimate stimuli elicited maximally distinguishable IT patterns (Ritchie, Tovar, & Carlson, 2015). Advancing this previous emphasis on the visual cortex, our cTBS data further show that the ATL is a key structure that supplements the computational ‘deficiency’ of the IT cortex, helping disambiguate two representationally-overlapping concepts.

Our findings lend further support to the emerging consensus that beyond visual cortices, neural processing of objects proceeds to the ATL, culminating in a high-level object representation that codes multimodal semantic identity rather than appearance (Lambon Ralph, 2014, Lambon Ralph et al., 2010, Patterson et al., 2007). Indeed, although the ATL has long been considered to be the apex of the visual ventral stream in visual neuroscience, there is now considerable convergent evidence that there is multimodal convergence of information in the ATL, which provides the basis for extraction of transmodal, coherent representations (e.g., Marinkovic et al., 2003, Shimotake et al., 2014, Visser and Lambon Ralph, 2011). Directly pertinent to and consistent with the current study, there is a potentially powerful convergence of results with previous neuropsychological data: SD patients with ATL atrophy tend to show an exaggerated typicality effect (e.g., while patients were able to select the correct colour for green vegetables they erred on most non-green vegetables by giving them green; Rogers et al., 2007). Close inspection of the current cTBS results (Fig. 5, Fig. 6A) shows that, in the conditions in which all unfavourable factors co-occurred (closely represented items, atypical target, brief display, and ATL stimulation), we observed the lowest accuracy amongst all conditions and much prolonged RT. This is consistent with the debilitating impact that ATL atrophy causes to the SD patients and supports the notion that the ATL structures hoard exemplar-specific information that is particularly needed when category-level information is overlapped and confusable.

A separate but relevant issue concerns the representational structure of different semantic categories – which we found in this study to be one of the important factors in successful rapid visual decisions. Together with previous studies using representational similarity analysis (RSA, e.g., Kriegeskorte et al., 2008) and hierarchical clustering (Hoffman & Lambon Ralph, 2013), the present TMS data support the conclusion that animal exemplars are represented more densely and overlapped less with artefacts and plants that tend to be coded more sparsely (see Rogers et al., 2004). It is possible that such representational distances among categories is partly driven by animacy (both plants and artefacts are inanimate entities; *cf.* Kriegeskorte et al., 2008). In addition to animacy, it might also involve differences in the functional dimension (edible plants for eating/cooking, tools for a particular usage, whereas animals may not always serve a specific functional purpose; *cf.* Hoffman & Lambon Ralph, 2013), as well as a denser representational packing for animals than other categories of objects (*cf.* Lambon Ralph et al., 2007, Rogers et al., 2004). It is important to note, however, that not all studies of semantic similarity have found this pattern. For example, Gainotti, Ciaraffa, Silveri, and Marra (2009, also see Gainotti, Spinelli, Scaricamazza, and Marra, 2013) had participants rate the saliency of different knowledge sources for objects (e.g., how salient is the colour aspect for the concept of ‘flamingo’); they found that animals and artefacts can be more similar to each other. Indeed, the exact relationships between these broad conceptual domains tend to vary systematically depending on which source of information is considered (*cf.* Hoffman & Lambon Ralph, 2013). We do not attempt to give a verdict on these hypotheses (e.g., animacy vs. functionality) as it is beyond the main focus of the present investigation. The key is the close alignment between the present cTBS data and previous investigations using feature-listing and RSA analysis, lending support to the conclusion that, during visual object recognition, top-down feedback hinges upon representational distance between object categories.

In the predictive coding hypothesis (Bar et al., 2006, Panichello et al., 2012, Trapp and Bar, 2015), the medial frontal cortices, particularly the OFC, has been suggested as the origin of top-down modulation. Some medial temporal areas, including the perirhinal and retrosplenial cortices, have been suggested to form a distributed network that works in tandem with the OFC to modulate the perceptual system. In the present work, we provide clear evidence that the ventrolateral aspect of the ATL is also a crucial component of the modulatory feedback system. A promising direction for future research would be to take a network approach, exploring how the ATL interacts with the medial frontal and temporal regions in generating semantically-based top-down feedback.

## Conflict of interest

The authors declare no competing financial interests.

## Footnotes

1 Before the pilot study that secured the paradigm we used in the present study, we first conducted a series of experiments to find the timeframes and presentation durations that permit optimal visibility and performance for the present study. Volunteers of the pilot study completed the same psychophysical procedure as those of the TMS study, except that they received no TMS. Results of accuracy showed a significant Duration × Typicality interaction (*F*_1,5_ = 7.91, *p* = .03) – the typicality effect (accuracy difference: typical minus atypical) was significantly greater for 40-msec displays (23%) than for 600-msec displays (5%). Results of reaction time also showed a marginally significant Duration × Typicality interaction (*F*_1,5_ = 6.34, *p* = .05) – the typicality effect (RT difference: atypical minus typical) was numerically greater for 40-msec displays (138 msec) than for 600-msec displays (46 msec). Together these suggest that participants were more susceptible to typicality during brief presentation.
